# Etymologia: *Nocardia*

**DOI:** 10.3201/eid2611.ET2611

**Published:** 2020-11

**Authors:** Christoffel J. Opperman

**Affiliations:** Groote Schuur Hospital, Cape Town, South Africa

**Keywords:** etymologia, Nocardia, bacteria, Edmond Nocard, filamentous, branching, genus, farcy, glanders

## *Nocardia* [no-kahr¢ e-əm]

The genus *Nocardia* is named in honor of Edmond Isidore Etienne Nocard (1850–1903), a French veterinarian and microbiologist who discovered the bacteria in 1888 from a bovine farcy case. He named this filamentous, branching bacteria *Streptothrix farcinica* (Greek *streptós*- “twisted” and *thrix* “hair”). Farcy (old French *farcin*), is a form of cutaneous glanders, characterized by superficial lymph node swelling and ulcerating nodule formation under the skin (Late Latin *farcīminum* “glanders,” from Latin *farcīmen* “a sausage,” from *farcīre* “to stuff”).

One year later, Trevisan characterized and termed the bacteria *Nocardia farcinica *(Figure), creating the genus *Nocardia*. In 1890, Eppinger isolated a similar organism from a brain abscess and called it *Cladothrix asteroides* (Greek *kládos*- “branch” and -*thrix* “hair”) because of its star-shaped colonies (Greek *asteroeidēs* “starlike”). Blanchard renamed the organism *Nocardia asteriodes* in 1896. Additional taxonomic work in 1962 resulted in *Nocardia asteroides* replacing *Nocardia farcinica* as the type species for the genus *Nocardia.*

**Figure:**
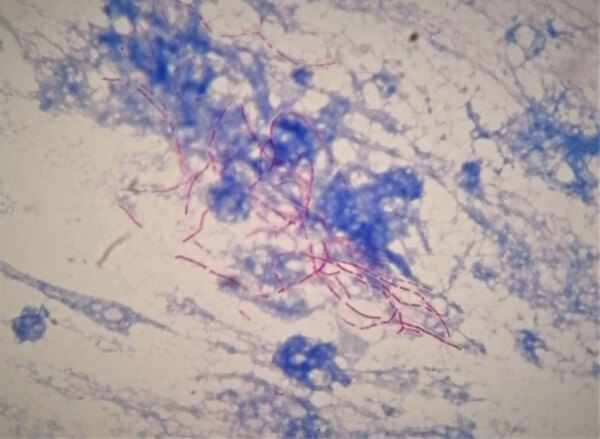
Twisted hair bacteria (*Nocardia* spp.) described by Edmond Nocard, from a bronchial alveolar lavage sample. Nocardiosis is an opportunistic infection, commonly associated with pulmonary disease. *Nocardia* are partially acid-fast, filamentous, branching bacilli (modified Kinyoun acid-fast stain using weak acid [0.5% sulfuric acid] for decolorization and methylene blue counterstain, original magnification x1,000.) Photograph courtesy of the author.
